# Supplementary material for: Antibiotic dose-response curves can measure antibiotic activity against Mycobacterium abscessus and Mycobacterium peregrinum
Source: bioRxiv. 2025 Dec 16:2025.12.16.694619. Preprint. [Version 1] doi: 10.64898/2025.12.16.694619 (PMC12724589; doi:10.64898/2025.12.16.694619)

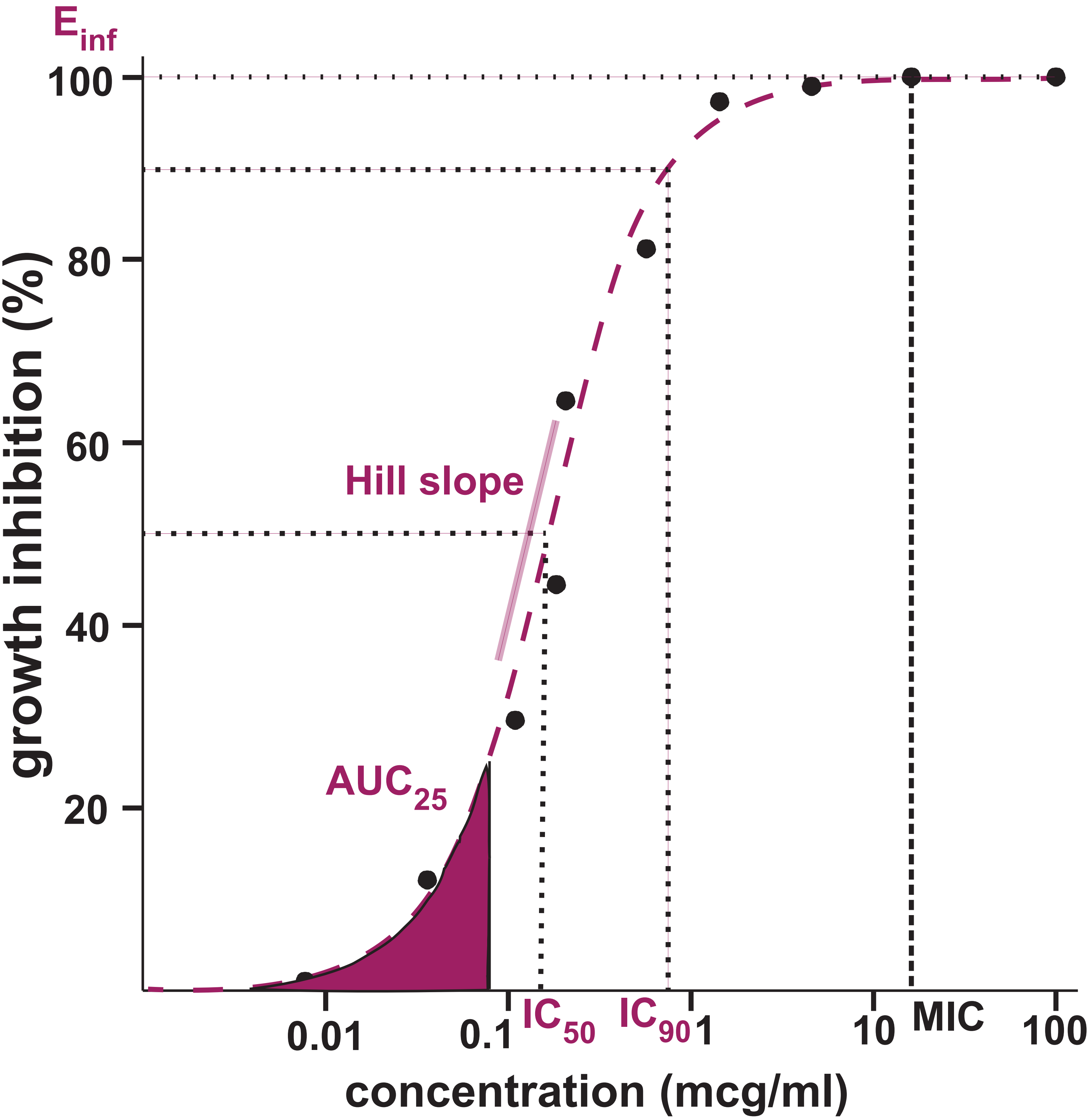

E_inf
100
80
60
40
20
Hill slope
AUC25
IC50
IC90
MIC
0.01
0.1
1
10
100
growth inhibition (%)
concentration (mcg/ml)

bioRxiv preprint doi: https://doi.org/10.64898/2025.12.16.694619; this version posted December 16, 2025. The copyright holder for this preprint (which was not certified by peer review) is the author/funder, who has granted bioRxiv a license to display the preprint in perpetuity. It is made available under aCC-BY 4.0 International license.

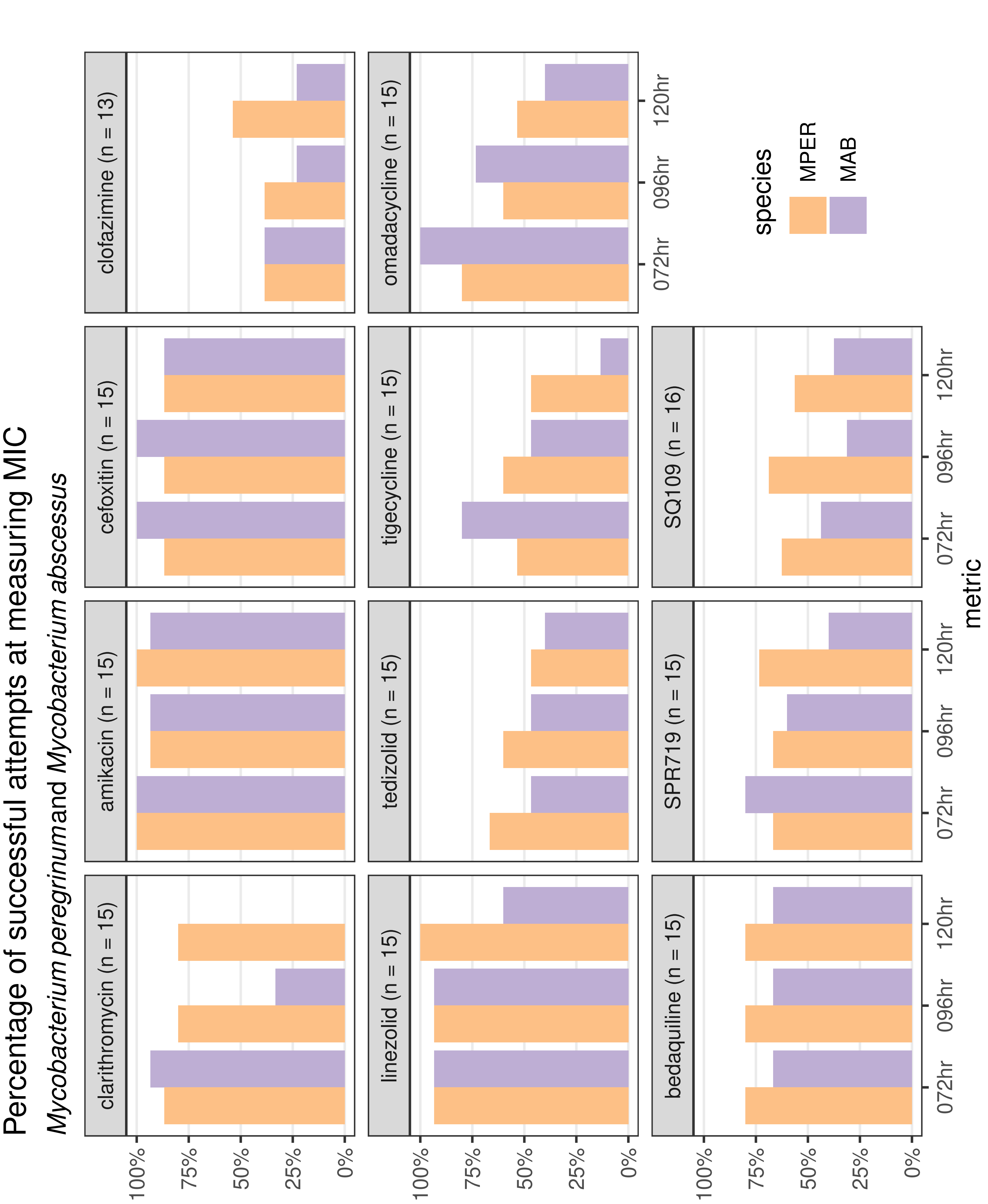

bioRxiv preprint doi: https://doi.org/10.64898/2025.12.16.694619; this version posted December 16, 2025. The copyright holder for this preprint (which was not certified by peer review) is the author/funder, who has granted bioRxiv a license to display the preprint in perpetuity. It is made available under aCC-BY 4.0 International license.

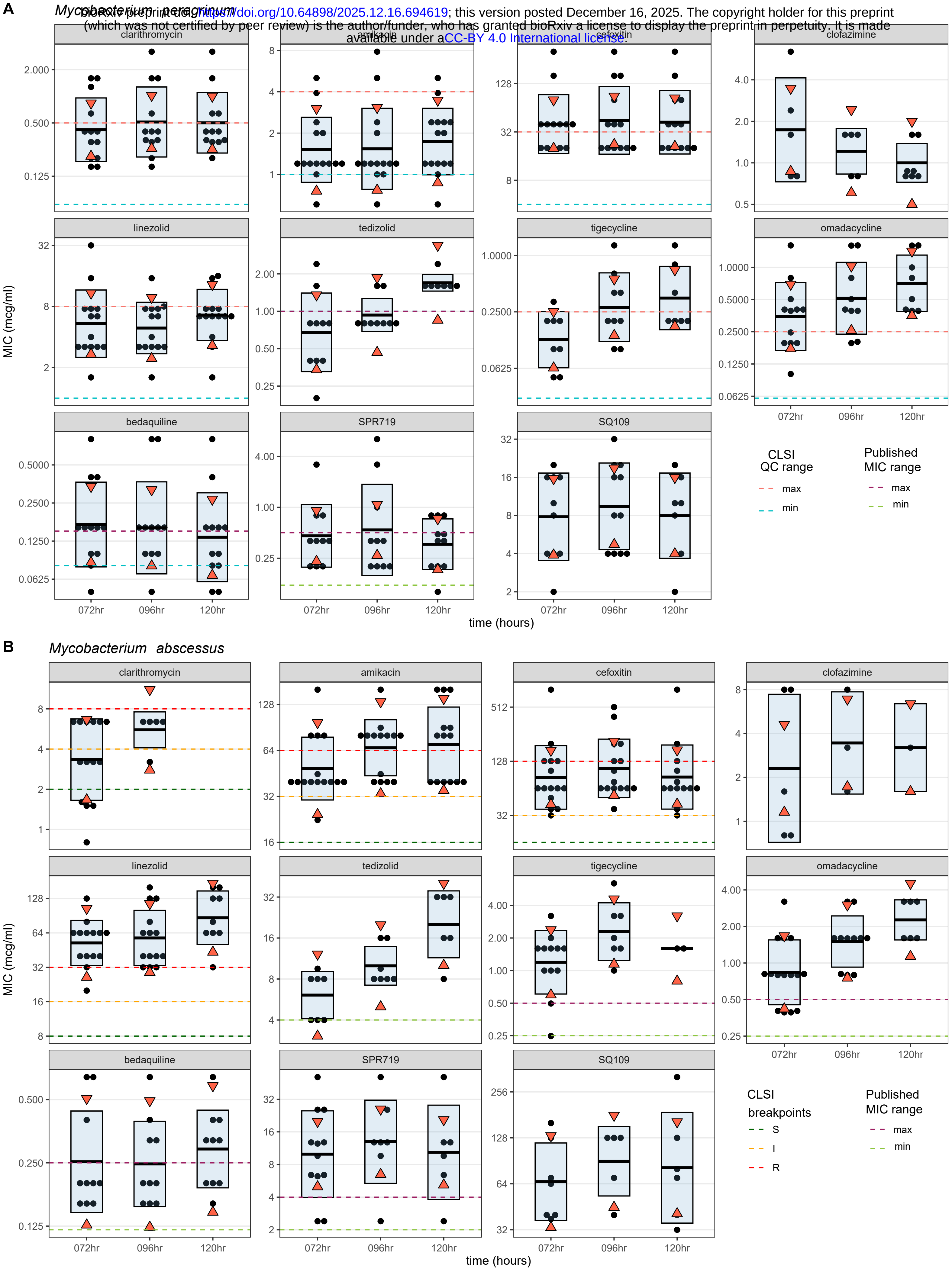

bioRxiv preprint doi: https://doi.org/10.64898/2025.12.16.694619; this version posted December 16, 2025. The copyright holder for this preprint (which was not certified by peer review) is the author/funder, who has granted bioRxiv a license to display the preprint in perpetuity. It is made available under aCC-BY 4.0 International license.

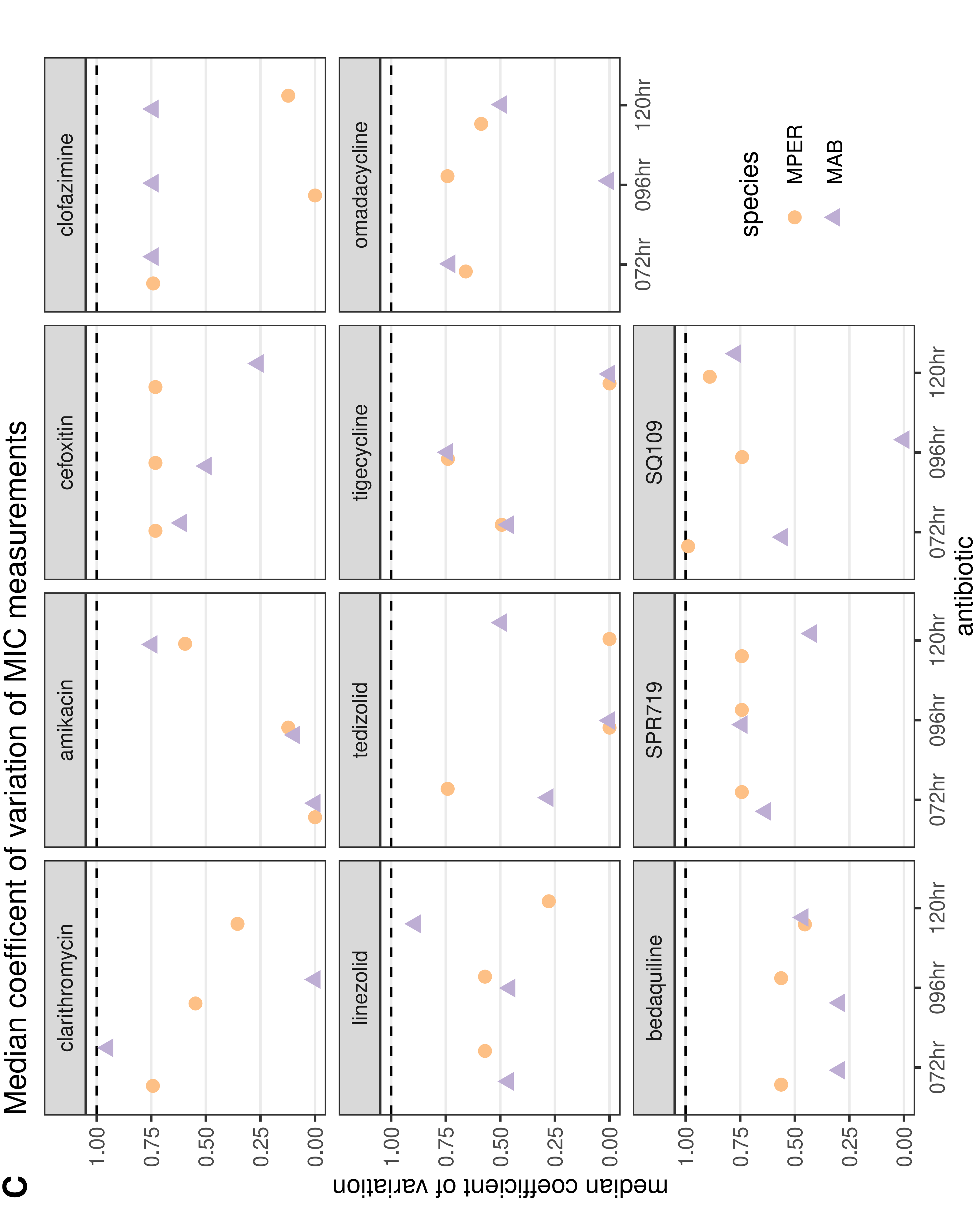

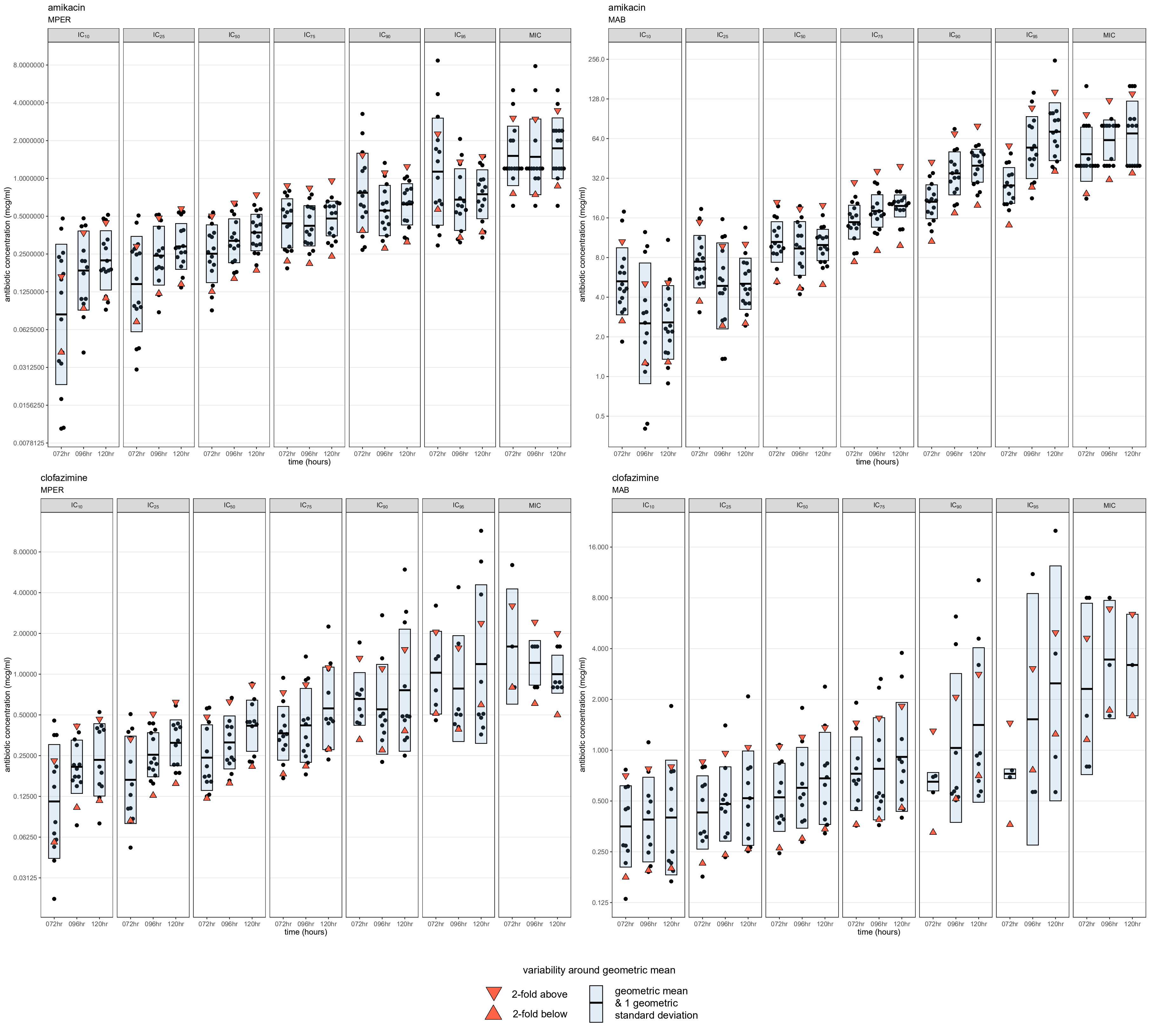

amikacin
MPER
amikacin
MAB
clofazimine
MPER
clofazimine
MAB
IC10
IC25
IC50
IC75
IC90
IC95
MIC
antibiotic concentration (mcg/ml)
time (hours)
072hr
096hr
120hr
8.0000000
4.0000000
2.0000000
1.0000000
0.5000000
0.2500000
0.1250000
0.0625000
0.0312500
0.0156250
0.0078125
256.0
128.0
64.0
32.0
16.0
8.0
4.0
2.0
1.0
0.5
8.00000
4.00000
2.00000
1.00000
0.50000
0.25000
0.12500
0.06250
0.03125
16.000
8.000
4.000
2.000
1.000
0.500
0.250
0.125
variability around geometric mean
2-fold above
2-fold below
geometric mean
& 1 geometric
standard deviation

bioRxiv preprint doi: https://doi.org/10.64898/2025.12.16.694619; this version posted December 16, 2025. The copyright holder for this preprint (which was not certified by peer review) is the author/funder, who has granted bioRxiv a license to display the preprint in perpetuity. It is made available under aCC-BY 4.0 International license.

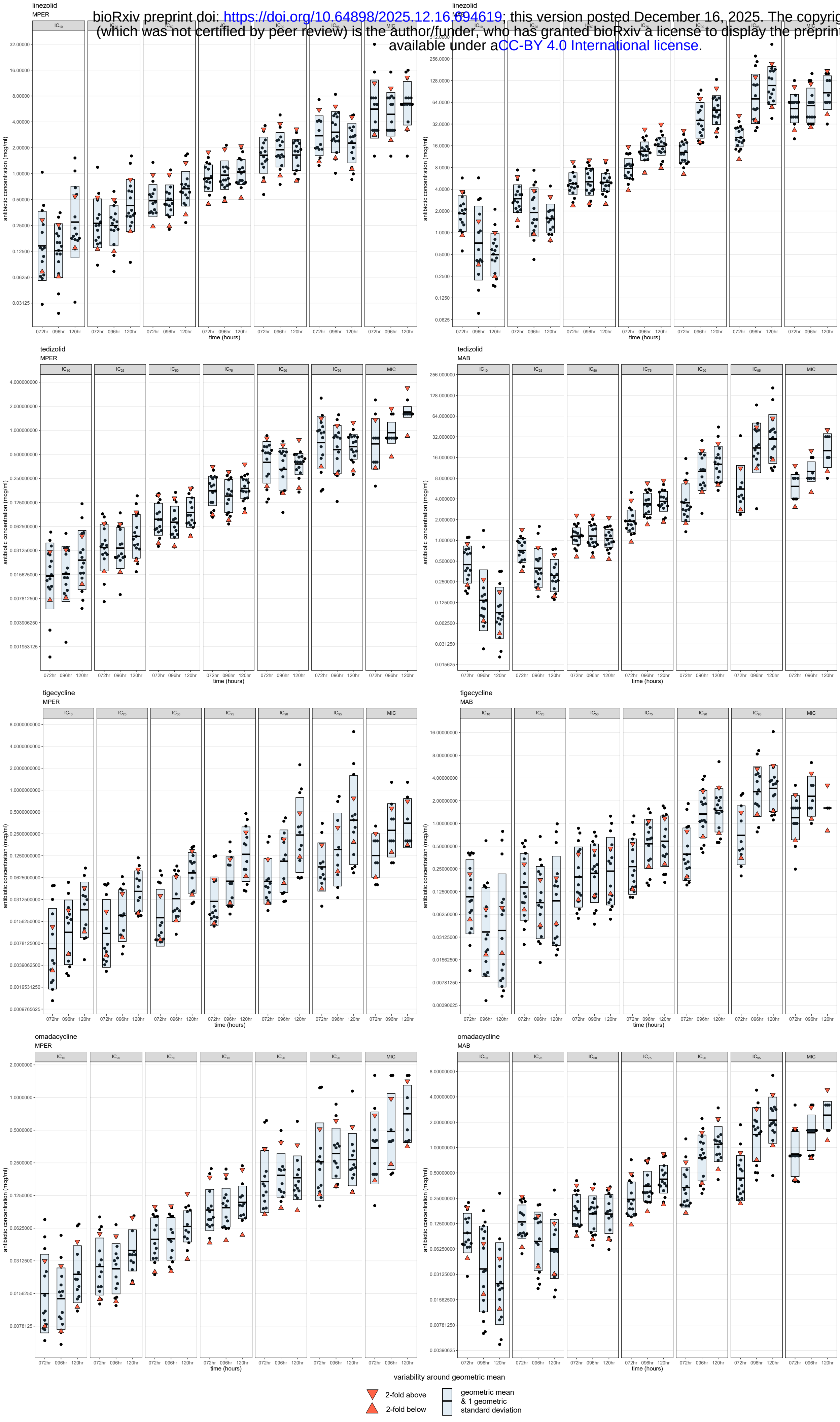

bioRxiv preprint doi: https://doi.org/10.64898/2025.12.16.694619; this version posted December 16, 2025. The copyright holder for this preprint (which was not certified by peer review) is the author/funder, who has granted bioRxiv a license to display the preprint in perpetuity. It is made available under aCC-BY 4.0 International license.

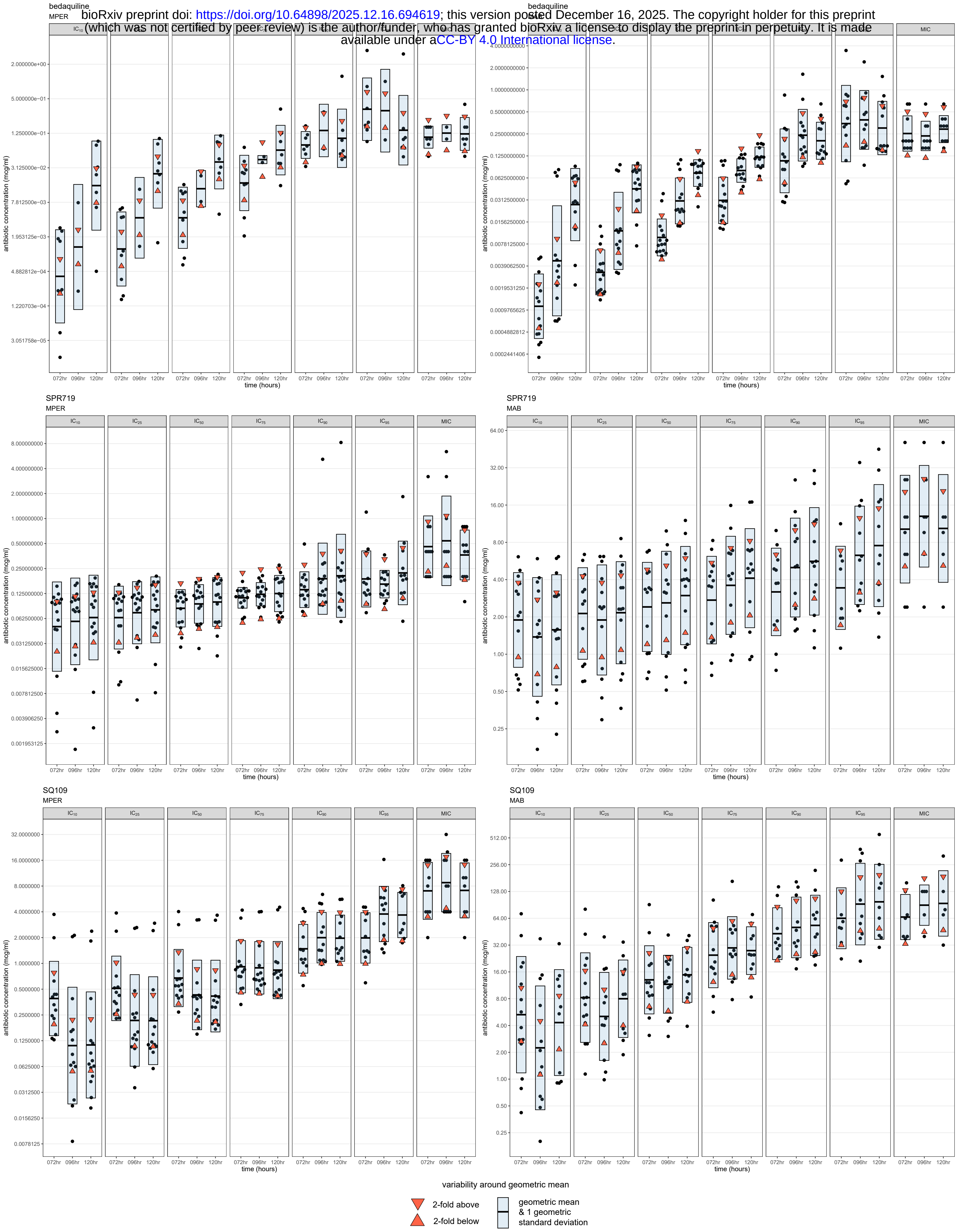

bioRxiv preprint doi: https://doi.org/10.64898/2025.12.16.694619; this version posted December 16, 2025. The copyright holder for this preprint (which was not certified by peer review) is the author/funder, who has granted bioRxiv a license to display the preprint in perpetuity. It is made available under aCC-BY 4.0 International license.

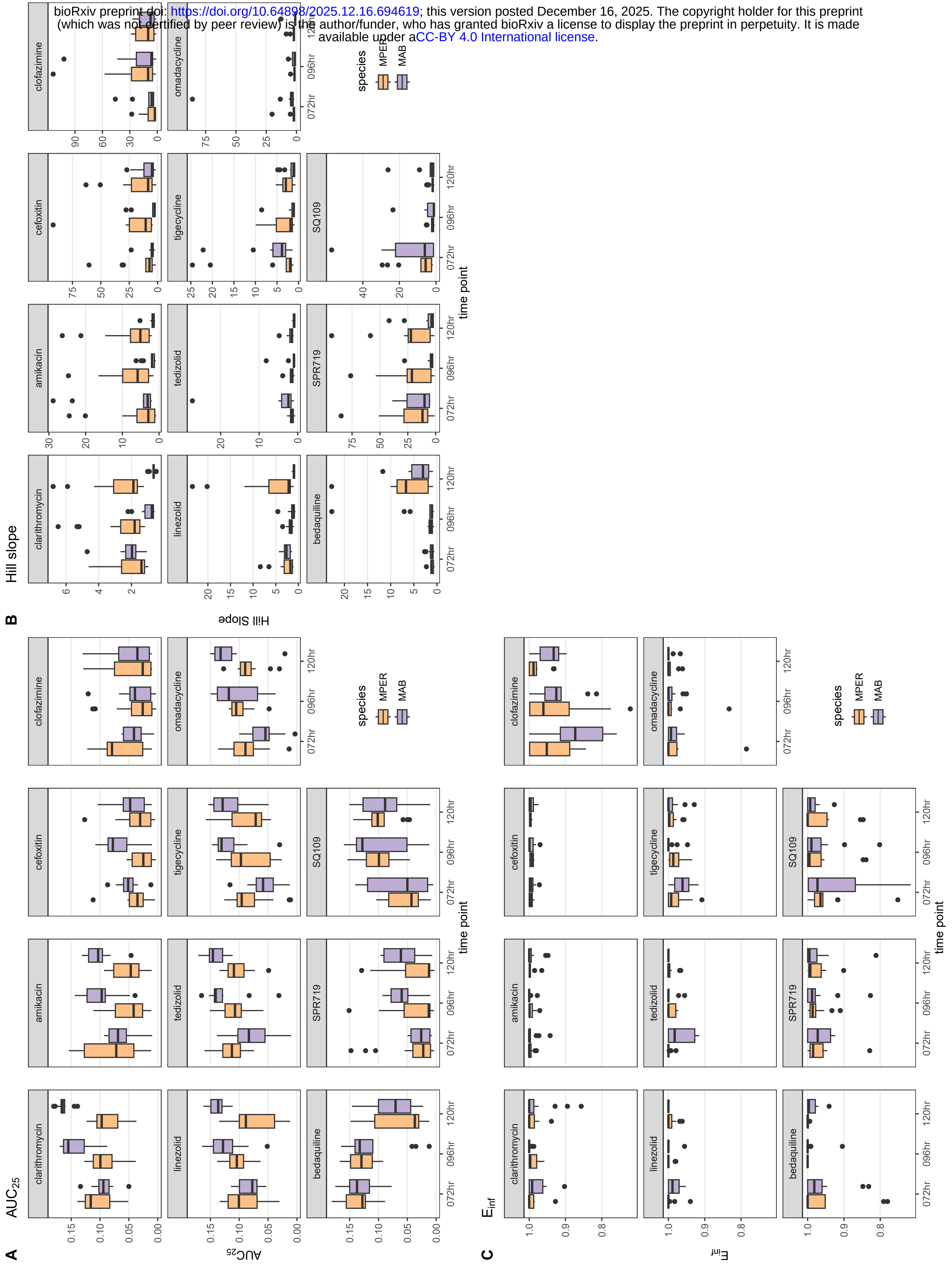

Supplement: Supplement 1 [file NIHPP2025.12.16.694619v1-supplement-1.pdf]
